# Conformational switch upon substrate binding informs the rational design of CCoAOMT enzymes

**DOI:** 10.1038/s41598-025-19623-1

**Published:** 2025-10-21

**Authors:** Yujie Cao, Xinru Yue, Wentong Yu, Pujin Yi, Yuxin Xiao, Liang Ma, Kaixuan Hu, Xin Fu, Jianping Hu, Xiang Nong, Wei Liu

**Affiliations:** 1 Bamboo & Forest Institute of Science, Technology and Industrial Innovation, Leshan Normal University, Leshan, 614004 China; 2 Key Laboratory of Medicinal and Edible Plants Resources Development of Sichuan Education Department, School of Pharmacy, Chengdu University, Chengdu, 610106 China; 3 College of Food Science and Pharmacy, Xinjiang Agricultural University, Urumqi, 830052 China

**Keywords:** Lignin, Caffeoyl coenzyme a o-methyltransferase, Caffeoyl coenzyme a, Molecular dynamics simulation, Protein design, Plant sciences, Ecology

## Abstract

Poplar is a deciduous tree belonging to the willow family, whose wood has the characteristics of light texture and toughness. It can be used to produce pulp, and is one of the main raw materials in the paper industry. A large amount of lignin is produced during pulping and papermaking process, where the generated waste liquid and pollutants are one of the main sources of industrial pollution. Regulating lignin content not only enables factories to process more pulp, but also greatly reduces the use of chemical reagents and the production of pollutants. Caffeoyl coenzyme A O-methyltransferase (CCoAOMT) is a class of S-adenosyl methionine (SAM) methyltransferases, which can effectively regulate the total amount of lignin biosynthesis. At present, molecular recognition mechanism of CCoAoMT with the substrate caffeoyl coenzyme A (CCoA) and corresponding conformational change both remain unclear, significantly restricting the design of weakly active enzymes. In this work, the overall conformational changes of CCoAOMT after binding CCoA were firstly explored by comparative molecular dynamics (MD) simulations. The results show that the four regions of α1, α2, α6 and α8 located outside binding pocket show greater flexibility, which is conducive to substrate binding. Through free energy landscape (FEL) and conformation cluster analyses, the representative functional motion modes were investigated, with the regions involved being β5, α2 and α8. According to adaptive steered molecular dynamics (ASMD) simulations, the complete recognition process of the receptor by SAM and CCoA, and allostery in the transport channel of substrates both were observed. With the binding of substrates, the structure of CCoAOMT gradually becomes more compact, accompanied by its transport channel from open to closed state. By binding free energy prediction and energy decomposition, a series of key residues favoring the receptor-substrate mutual recognition were proposed. Specifically, CCoA exhibits H-bonds with N170, D218, T42, Y188, N39, R186, N174 and R30, as well as hydrophobic interactions with K1 and I40. In particular, Y188 and D218 both anchor CCoA caffeoyl group to the Ca^2+^ active site by forming stable H-bonds. Based on exhaustive single mutation and folding entropy calculation, six mutants (K1L, N39G, I40P, T42N, N174D and D218V) may be candidates for low-activity CCoAOMT.

**Supplementary Information:**

The online version contains supplementary material available at 10.1038/s41598-025-19623-1.

## Introduction

 Poplar is a kind of deciduous tree belonging to the willow family, which has the characteristics of rapid growth, strong adaptability, cold and drought tolerance^1^. Its wood is light and tough, suitable for making furniture, pulp, etc. The history of poplar papermaking can be traced back to ancient China, where Cai Lun in the Han Dynasty invented the world’s first paper in 105 BC using fibers from plants such as poplar, mulberry and hemp^2^. This invention greatly changed the way humans wrote and recorded, and also widened the utilization of paper around the world. Poplar (Populus spp.) remains a predominant raw material in the pulp and paper industry because of its favorable fiber morphology and rapid growth cycle^3,4^. Advancements in paper manufacturing technologies have expanded the functional applications of paper-based products. These developments present concurrent challenges and opportunities for innovation in sustainable resource utilization and environmental stewardship, particularly in waste management, energy efficiency, and circular economy implementation. The papermaking is one of the core consumer industries of wood, while bring serious environmental pollution in China^5–7^. The biggest sources of pollution in the poplar papermaking process mainly include chemical use, water resource consumption and pollution, energy consumption and air pollution, solid waste and so on. The wastewater discharge and chemical oxygen consumption (COD) in paper industry account for 15–20% and 10–15% of the total industrial emissions in China, respectively. In the process of pulping, chemicals are usually used to separate the lignin and cellulose in raw wood, not only producing cellulose-rich pulp but also bringing a large amount of waste liquid for the treatment of lignin^8–10^.

Lignin is a class of phenolic secondary metabolites with three different structural categories: lilac-based lignin (S-lignin), wood-based lignin (G-lignin) and hydroxyphenyl lignin (H-lignin). Lignin plays an important physiological function in plants^11–14^ and can attenuate the decomposition of cellulose by cellulolytic enzymes. Sulis et al., found that reducing the Lignin content in wood at the level of gene regulation could enable the factory to process 40% more pulp, not only producing more fiber but also greatly reducing pollutants^15^. Due to complexity and diversity of lignin structure, its biosynthesis pathway is mainly divided into three stages: phenylpropane pathway, monomer methylation reaction, and monomer polymerization reaction^16^. Among them, the phenylpropane pathway is not specific also occurring in the synthesis of other secondary metabolites^17^. Cinnamyl coenzyme A reductase (CCR) and cinnamyl alcohol dehydrogenase (CAD) both were involved in monomer polymerization reaction. Even if the expression of CAD and CCR genes was inhibited, structural components and total content of lignin still did not change significantly^18^. Nevertheless, the absence of caffeoyl coenzyme A O-methyltransferase (CCoAOMT) and catechol O-methyltransferase (COMT) during lignin synthesis definitely inhibits the methylation of lignin monomers^19^.

CCoAOMT is a bisubstrate protein that can bind either S-adenosyl methionine (SAM) or caffeoyl coenzyme A (CCoA). Structurally, CCoAOMT consists of 7 hydrophobic Rossmann-like β-sheets, 8 amphipathic α-helices and a series of loops^20^. Zhao group carried out real-time fluorescence quantitative polymerase chain reaction (PCR) to explore expression patterns of CCoAOMT gene subtypes in different plant tissues under different nitrogen forms and concentrations^21^. It is found that nitrogen has a significant impact on lignin content in plants. Ji analyzed the preliminary heterologous expression in transgenic *Arabidopsis thaliana* by the wet chemistry and gas chromatography mass spectromete (GC-MS) experiments^22^. The results showed that down-regulation of CCoAOMT significantly reduced Lignin content by 24%, while its over-expression resulted in a 40% increase. By measuring expression level of target gene, lignin contents, lignin monomer, cellulose, and stem anatomical structure in transgenic tobacco lines, Xiao et al. found that low expression of CCoAOMT significantly inhibited the synthesis of G-lignin^23^. Wagner et al. also found that low-activity CCoAOMT could effectively reduce the lignin content by studying *Pinus radiata*^24^. By using confocal Raman mapping (CRM) and liquid chromatograph mass spectrometer (LC-MS) to analyze the lignin formation and metabolism in *Catalpa bungei* induced by artificial bending, Yao et al. found that CCoAOMT has a high level of transcription and translation, which may be the key to the exception of lignin synthesis^25^. Through isothermal titration calorimetry (ITC) experiment, Alexander et al. found that CCoAOMT showed a sequential recognition mechanism, that is, SAM binding was earlier than CCoA; The Ca^2+^ ion in pocket promotes the deprotonation of CCoA by coordinating the active hydroxyl group^26^.

The scientific conclusion that regulating CCoAOMT activity can effectively determine the total amount of lignin biosynthesis has been widely accepted^27^. In terms of biological function, CCoAOMT is one of the key enzymes in the lignin biosynthesis pathway, taking CCoA as substrate and transferring methyl group on SAM to the C3 position at benzene ring of lignin monomer^28^ to form feruloyl coenzyme A. The CCoAOMT protein is composed of two subtypes—CCoAOMT1 and CCoAOMT2. Natraraj et al. evaluated protein models through PROCHECK, WHATCHECK, Verify3D and ERRAT programs, proving that CCoAOMT2 was superior to CCoAOMT1 in embodying active conformation^29^. Here, the CCoAOMT2 with active conformation was determined as the investigated object, uniformly represented by CCoAOMT for analysis convenience in this work. As an indispensable synthetic precursor of lignin monomer, CCoA also participates in subsequent polymerization reaction.

**Fig. 1:**
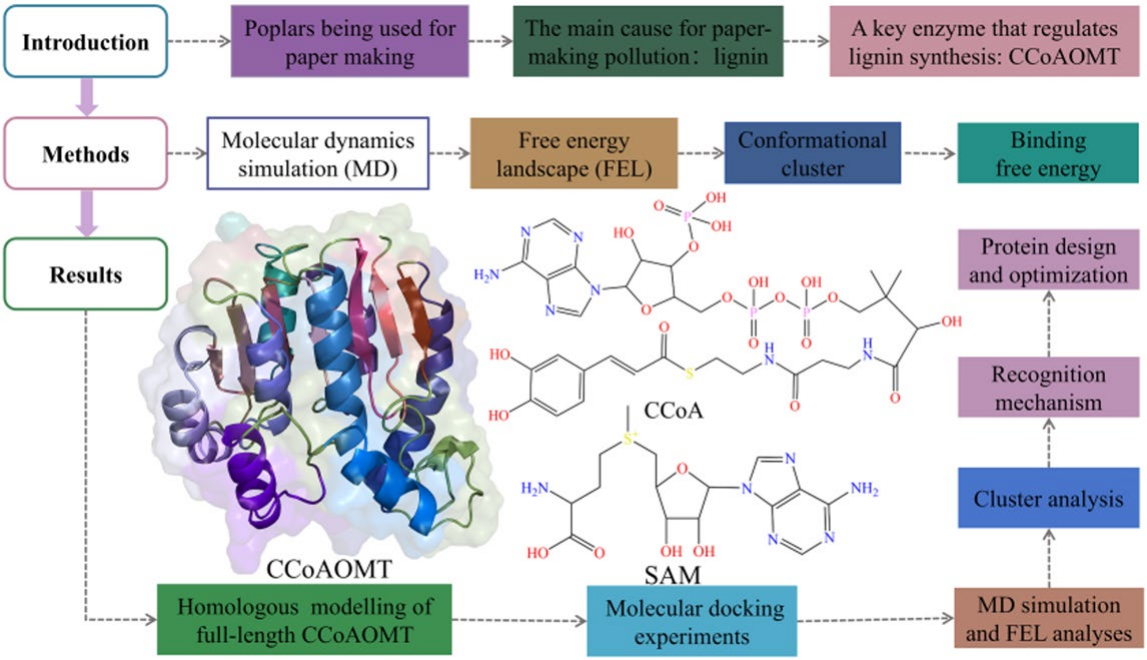
Research protocol of this work, as well as structures of CCoAOMT protein and its double substrates (i.e., CCoA and SAM).

There have been a series of plant physiological, biochemical, and structural biology experiments previously, elucidating structure-function relationship of CCoAOMT. Nevertheless, molecular recognition with substrate CCoA and mechanism-based low-activity CCoAOMT design both have not been reported. In this work, comparative molecular dynamic (MD) simulations were performed for CCoAOMT monomer and its complex with CCoA (see Fig. 1). According to free energy landscape (FEL) and conformation cluster analyses, structural changes of CCoAOMT after binding CCoA and the open-close state in substrate transport channel both are deeply investigated. Moreover, binding free energy calculation was also carried out to determine the main driving force of receptor-ligand complex formation. Based on energy decomposition, key residues favoring molecular recognition were mined. Finally, a possible design strategy of low-activity CCoAOMT was proposed through exhaustive residue mutation, binding free energy and folding free energy calculation.

## Methods

### Molecular Docking

ChemBio3D Ultra 12.0 (Cambridge Soft, Cambridge, MA, USA) was used to construct molecular structures of CCoA, and the initial protein structure was derived fromcrystal structure (PDB ID: 5KVA). Subsequently, energy optimization was performed for these structures under the MM2 molecular force field with the convergence criterion of energy root mean square (RMS) less than 0.0001 kcal·mol^−1^·Å^−2^. The geometry optimized structure was imported into SYBYL-X 2.0, and molecular charge was calculated by Gasteiger-Huckel method; based on Tripos force field, the 2nd energy optimization was carried out with convergence criterion of energy RMS less than 0.005 kcal·mol^−1^·Å^−2^. After two steps of minimization, the small molecule structure reaches the lowest energy, indicating that the structure meets the requirements of initial ligand conformation for subsequent molecular docking^30^.

Molecular docking serves as an important supplement to structural biology experiments, predicting the binding patterns of receptor with ligand via computer simulations. In principle, the complex modes by molecular docking needs to achieve energy matching and geometric matching at the binding site^31,32^. Here, the small molecule CCoA was bound to the active pocket of CCoAOMT using the AutoDock 4.0 package^33,34^, and the atomic charges of the ligand CCoA were assigned using the Gasteiger-Hückel charge model, which is integrated into the AutoDock 4.0 package and widely applied in molecular docking studies for small molecules with similar chemical properties. This charge model provides a reasonable approximation of the electrostatic interactions between the ligand and receptor, consistent with the parameters of the Lamarckian genetic algorithm (LGA) used in our docking simulations. In AutoDock, Lamarckian genetic algorithm (LGA) and local search (LS) strategy both were adopted to find the suitable conformation of the receptor-ligand complex.

In this study, the size of docking box was set to 40 Å × 40 Å × 40 Å, with the spacing between grid points being 0.375 Å and box center coordinates being (53.825, 32.200, 66.230), We first searched for the three important amino acids above the binding pocket using the Cα atoms of these three amino acid residues to characterize their positions and then calculated the geometric center of these three points as the center of the docking box. Docking times were set to 128, and the snapshot with the lowest energy in the maximum cluster was determined as the optimal near-natural complex model. Based on molecular docking experiment, the complex model of the SAM-bound activated CCoAOMT with CCoA is obtained (i.e., CCoAOMT*_CCoA), where the asterisk in the upper right corner is used to represent activated state of the receptor. The determination of complex models aids to understand the structure-function relationship and ligand recognition details of CCoAOMT at the atomic level.

### Molecular dynamics simulations

Based on Newtonian mechanics, molecular dynamics (MD) simulation has been widely utilized to explore molecular interactions, motion modes and thermodynamic/kinetic properties of protein receptors with ligands^35,36^. In this work, comparative MD simulations were performed for the two systems: CCoAOMT* (i.e., the CCoAOMT being bound and activated by SAM) and CCoAOMT*_CCoA (i.e., the ternary complex of CCoAOMT, SAM and CCoA). Triplicate repeated simulations were performed for each system. The results demonstrated high consistency across replicas (backbone RMSD < 0.2 Å; free energy SD ≤ 0.6 kcal·mol^−1^). Consequently, one representative experiment was selected for subsequent mechanistic analysis to balance the computational efficiency and statistical robustness. Before MD simulations, all the force field parameters of Ca2 + bonding and the adjacent residues were constructed by MCPB.py methods, where the force field parameters were calculated by the Seminario method^37^. The involved quantum mechanical calculations, such as geometric energy optimization and Merz-Kollman charge, are done in Gaussian 09^38^. In this work, the atomic type calculations were performed using the B3LYP hybridization generalization and the 6-31G* basis group^39^. To reduce the unreasonable atomic collisions in the simulation systems, a solute-constrained geometric optimization is performed with 5000 steps of steepest descent (SD) and 5000 steps of conjugate gradient (CG) energy minimization (EM), where the constraint constant is set to 500 kcal·mol^−1^·Å^−2^. It is followed by solute-unconstrained geometric optimization including 20,000 steps of SD and 20,000 steps of CG EM, with the convergence criterion of energy gradient less than 0.01 kcal·mol^−1^·Å^−2^.

After the above two EM steps, the following MD simulations were carried out using AMBER20 software package and ff14SB force field^40^. MD simulations were also divided into two stages: (1) under the canonical ensemble with volume and temperature (NVT) a 5 ns solute-constrained one with a force constant of 10 kcal·mol^−1^Å^−2^, where temperature gradually increased from 0 to 300 K; (2) another 495 ns solute-unconstrained productive stage, adopting SHAKE algorithm to adjust the hydrogen-containing atoms. In the whole MD simulation process, 11,446/11,457 water molecules were respectively added to the CCoAOMT* and CCoAOMT*_CCoA systems by TIP3P water model. Moreover, four Na + ions are also added to both systems to ensure the environmental neutrality, being conducive to the smooth simulation. Non-bonded interaction radius and integration step were set to 10 Å and 2 fs, respectively. During MD simulations, the conformational fluctuation was monitored by VMD 1.9.3 package. Atomic coordinates were stored every 5 ps, and a total of 10,000 snapshots were collected over a 500 ns production run for the subsequent analyses of molecular recognition and conformational change.

### Free energy landscape

By comparing the free energy basin and free energy barrier, free energy landscape (FEL) can be used to analyze molecular motion and conformational change for the CCoAOMT* and CCoAOMT*_CCoA systems. It helps to understand their biological processes such as molecular recognition, protein folding and aggregation^41^. The free energy basin is composed of a series of steady-state conformations for biomolecules that can be identified by different ligands under physiological conditions. The free energy barrier represents the transient state between various steady-state conformations. The required conformational sampling for FEL analysis was achieved based on principal component analysis (PCA) of MD trajectories^42^. The first (PC1) and second principal components (PC2) both serve as reaction coordinates for FEL mapping. According to the ensemble theory in statistical mechanics, the higher the probability proportion of a certain conformation is, the lower its folding free energy. The folding free energy ∆*G*(*X*) can be calculated as follows:

1 $$\:\varDelta\:G\left(X\right)=-{k}_{\text{B}}T\text{l}\text{n}P\left(X\right)$$

where *X* represents one of the principal components (PCs) of MD trajectories; *P(X)* refers to the conformational distribution probability of the system along this PC motion, representing the contribution to the overall PCs; *k*_*B*_ and *T* are the Boltzmann constant and absolute temperature, respectively. In a word, FEL analysis based on PCA dimension reduction can provide structural differences and transitions at the functional motion level.

### Conformation cluster analysis

Conformation cluster analyses were carried out for the 10,000 snapshots in the CCoAOMT* and CCoAOMT*_CCoA systems with MMTSB software package^43^. The basic idea of conformation clustering was based on the peptide folding work by Daura^44^. The root mean square deviation (RMSD) of Cα atoms between different snapshots is first calculated one by one, and the RMSD matrix (N × N, N is the total snapshot number) is then established. If the RMSD difference between any two snapshots is less than the preset threshold value, they are assigned to a specific cluster (see Formula 2).

2 $$\:C=\left\{\begin{array}{c}1\:if\:\varDelta\:RMSD\le\:1.7\: \text{\AA} \\\:0\:if\:\varDelta\:RMSD>1.7\: \text{\AA} \:\end{array}\right.$$

In this study, when ∆RMSD is less than the threshold value of 1.7 Å, C equals 1, indicating that the two snapshots belong to the same cluster. If C is equal to 0, they belong to different clusters. For the remaining snapshots that have not yet been clustered, the same operation is continued until all conformations are grouped. Significantly, CCoAOMT has relatively rigid β-sheets (β4, β5) at the Ca²+ center and more flexible α-helices (α1, α2, and α8) at the periphery. The 1.7 Å threshold effectively captured conformational differences in the flexible regions while accounting for the stability of the rigid core, ensuring that the clusters reflected biologically relevant functional states. In addition, the snapshot with the lowest energy is designated as the representative conformation for each cluster. In fact, cluster analysis can be used for effectively excavating the potentially functional regions with the greatest conformational differences. It can also successfully be applied to the comparison among some local structural features and parameters.

### Binding free energy calculation

As an important physicochemical parameter to characterize receptor-ligand affinity, binding free energy has become the key index to evaluate drug activity^45^. In this work, binding free energy of CCoAOMT* with CCoA and its driving forces both are predicted with molecular mechanics/Poisson-Boltzmann solvent area (MM/PBSA) methods^46,47^, In our binding free energy calculations, we employed the implicit solvation model. Specifically, for the MM/PBSA method, the Poisson - Boltzmann equation was used to calculate the polar solvation free energy. In this process, the protein and ligand were treated as charged particles, and the solvent was simulated as a continuous uniform medium. To accurately represent the dielectric environment, we set the interior dielectric constant of the solute (Din) based on previous research in this field. For the systems studied, a value of 2.25 was chosen for Din in the SIE method calculations, which is a commonly used value for similar systems and has been demonstrated to provide reasonable results in representing the electrostatic interactions within the solute. This value was also applied when considering the polar part (ELEPB) in the MM/PBSA calculation. Next, binding free energies of a series of CCoAOMT* mutants to CCoA were also calculated with solvated interaction energy (SIE) method, aiding to gain the potential low-activity CCoAOMT enzymes.

One snapshot was picked up every 8 ns from the relatively stable 300–500 ns MD trajectories, with total 25 samples being collected. In the MM/PBSA calculation, binding free energy ($\:\varDelta\:{G}_{\text{c}\text{a}\text{l}}$) can be divided into two energy terms—enthalpy change (∆*H*) and temperature product of entropy change (*T*∆*S*). The former is composed of vacuum molecular internal energy (∆*E*) and solvation free energy (Δ*G*_sol_). The binding free energy by MM/PBSA is calculated as follows:

3 $$\:\varDelta\:{G}_{\text{c}\text{a}\text{l}}=\varDelta\:H-T\varDelta\:S=\varDelta\:E+\varDelta\:{G}_{\text{s}\text{o}\text{l}}-T\varDelta\:S=\left({ELE}_{\text{I}\text{N}}+{VDW}_{\text{I}\text{N}}+{ELE}_{\text{P}\text{B}}+{VDW}_{SA}\right)-T\varDelta\:\text{S}$$

Specifically, ∆*H/* ∆*G*_sol_ both are composed of the polar (*ELE*_IN_/*ELE*_PB_) and non-polar (*VDW*_IN_/*VDW*_SA_) parts. *T* is the absolute temperature in Kelvin. Entropy change (i.e., ∆*S*) characterizes the disorder of receptor folding conformation after recognition by the ligand, calculated with normal mode method. Before ∆*S* is calculated, the snapshots are respectively geometric optimized for 10^5^ steps of steepest descent (SD), with the convergence condition of energy gradient being 0.0001 kcal·mol^−1^·Å^−2^.

The SIE method can also be used to evaluate the affinity and stability of the receptor-ligand complex by calculating intermolecular interaction energy. It has been widely used in protein design because of high efficiency and accuracy. The binding free energy, $\:\varDelta\:{G}_{\text{b}\text{i}\text{n}\text{d}}$ (*ρ*, *D*_in_, *α*, *γ*, *C*), by SIE is calculated as follows:

4 $$\:\varDelta\:{G}_{\text{b}\text{i}\text{n}\text{d}}(\rho\:,{D}_{in},\alpha\:,\gamma\:,C)=\alpha\:\left[{E}_{c}\right({D}_{in})+{\Delta\:}{G}_{\text{bind}}^{R}(\rho\:,{D}_{in})+{E}_{vdw}+\gamma\:{\Delta\:}MSA(\rho\:\left)\right]+\text{C}$$

where the parameter α represents the global scale factor to characterize conformational entropy loss with the default value of 0.1048. The *E*_c_ (*D*_in_) is the coulomb interactions, where *D*_in_ is the internal dielectric constant of solute set at 2.25. The $\:{\Delta\:}{G}_{\text{bind}}^{R}(\rho\:,{D}_{in})$ represents the change of the reaction field energy, which is calculated by the boundary element program BRI BEM. Here, ρ is the Linear proportional constant of van der Waals radius with default value of 1.1. The *E*_vdw_ represents van der Waals interactions. The parameter *γ* is the surface area coefficient set to 0.0129 kcal·mol^−1^·A^−2^; the Δ*MSA*(*ρ*) indicates the change in surface area. The parameter *C* is calibration constant with the default value of −2.89 kcal·mol^−1^.

### Adaptive steered molecular dynamics (ASMD) simulation

Steered molecular dynamics (SMD) simulations are designed to reproduce the principles of atomic force microscopy (AFM) experiments. During the SMD simulation, the investigated system is pulled by a cantilever, and an external force is applied in a given direction to accelerate the dissociation/binding of receptor-ligand or conformational change of biological macromolecules^48–50^. As the most common way to describe the motion of periodic bound states, the harmonic potential energy function U is defined as follows:

5 $$\:\text{U}=\frac{1}{2}\text{k}{\left[\text{v}\text{t}-(\text{X}\left(\text{t}\right)-\text{X}(0\left)\right)\cdot\text{n}\right]}^{2}$$

where k, v, t, n are respectively used to represent elastic coefficient, motion velocity, motion time and the unit direction vector. X(t) and X(0) are the coordinates of mass center for the pulled molecule at times t and 0. Indeed, the unchanged artificial pulling direction is likely to deviate from natural dissociation pathway of the complex. In some cases, it will lead to an error in the estimation of dissociation energy and even a failure of the dissociation simulation. As shown in Eq. 6, the spring force can be obtained by multiplying the elastic coefficient k with the coordinate deviation value:

6 $$\:\text{F}(\text{X},\text{t})=\text{K}\left(\text{X}\right({\text{t}}_{0})-\text{X}(\text{t}\left)\right)$$

In addition, the Eq. 7 shows the connection between the potential energy U and the force F(X, t), that is, the force F(X, t) can be gained from the partial derivative of the potential energy U with respect to the generalized coordinates X.

7 $$\:\frac{\text{d}\text{U}}{\text{d}\text{X}}=\text{F}(\text{X},\text{t})+{\upsigma\:}\text{N}\left(\text{t}\right)-{\upgamma\:} v$$

where σN(t) is the random fluctuation term with average value of 0, and σ refers to the amplitude. The last term $\:{\upgamma\:}$ν is the friction force, which can be expressed by the product of a nonlinear velocity ν and the friction coefficient γ.

Since the dissociation rupture force is closely related to binding free energy, ASMD treats pulling force as an indication of the unbinding free energy barrier, and constantly sets motion direction to correct the defect caused by the fixed unit direction vector^51–53^. According to the Jarzynski equality concerning the non-equilibrium approximation, the free energy difference between two equilibrium states can be calculated by the exponential averaging of generalized work. The Eq. 8 gives the free energy difference (Δ*G*) calculation method based on non-equilibrium work (*W*):

8 $$\varDelta\:{\text{G}}\: =- \frac{1}{{\upbeta}}\sum_{\text{i}=1}^{\text{N}'}\text{ln}{\langle{e}^{{-\beta\:W}_{{{\upxi\:}}_{\text{i}}\leftarrow{{\upxi\:}}_{\text{i}-1}}}}\rangle_{\text{i}-1}$$

where β is equal to 1/(k_B_T), k_B_ and T are Boltzmann’s constant and absolute temperature, respectively.〈.〉_i−1_ denotes an ensemble average over the non-equilibrium trajectories. In ASMD algorithm, the overall reaction coordinate ξ is partitioned into N′ segments (i.e., ξ_0_, ξ_1_, …, ξ_N′_). The work *W*_ξi_ of each segment is calculated separately, and the total work (corresponding to free energy change ΔG_ξ1←ξ0_) is obtained by summing. The stretching force is minimized based on genetic algorithm (GA), and a dissociation path with a lower energy barrier is obtained by automatically adjusting the stretching direction^54,55^.

To investigate the binding process of SAM and CCoA, the free energy changes along the binding path were calculated based on the specific structures obtained from ASMD simulations. The distances travelled by SAM and CCoA from the outside to the inside of cell membrane was determined as the reaction coordinate. According to the changes of entry channels provided by the HOLE analysis^56^, the entire dissociation route was divided into five segments with a pull-up time of 40 fs each. In the ASMD simulations, specific atom pairs were constrained to drive conformational sampling: Protein-ligand pair 1: Cα atom of Val68 in CCoAOMT and C1 atom of SAM and Protein-ligand pair 2: Cα atom of Asp46 in CCoAOMT and C1 atom of CCoA, which is used to character the dissociation of SAM with CCoA from their corresponding pockets. In addition, the constant traction speed and the harmonic force constant k are respectively set to 1 Å·ns^−1^ and 2.5 kcal·mol^−1^Å^−2^, with a total of 25 tracks per segment collected to obtain statistically reliable results. All ASMD simulations were performed using PMEMD embedded in the AMBER20 software package, with data acquisition every 2 fs in the NVT canonical ensemble. Based on the Langevin thermostat at 300 K, the mean force potential (PMF) was calculated via umbrella sampling by the WHAM module.

## Results and discussion

### Structural rationality of Poplar CCoAMOT model

**Fig. 2:**
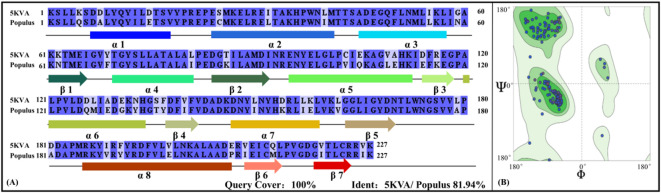
Sequence alignment and Ramachandran plot of poplar CCoAMOT model.

So far, four CCoAOMT crystal structures have been collected in the protein database (PDB), respectively from *Sorghum bicolor* (PDB ID: 5KVA), *Synechocystis* (PDB ID: 3CBG) and *Medicago sativa* (PDB IDs: 1SUI and 1SUS)^57^. Only in the 5KVA structure does the unmethylated SAM appear. Here, a sequence alignment was performed between 5KVA (i.e., CCoAOMT from *Sorghum bicolor*) and poplar CCoAOMT (see Fig. 2A). There was a high sequence similarity (~ 81.94%) between the two CCoAMOT systems with the coverage of 100.0%, indicating that the corresponding genetic relationship was very close. Additionally, among the available CCoAOMT structures (5KVA, 3CBG, 1SUI, and 1SUS), 5KVA exhibits the highest resolution of 1.827 Å, significantly outperforming 3CBG (2 Å), 1SUI (2.7 Å), and 1SUS (2.7 Å). This high-resolution template ensured the accuracy of key structural features, particularly in the active pocket and substrate-binding regions, which are critical to our analyses. Therefore, the 3D structure of poplar CCoAOMT was constructed based on the Swiss-model server using 5KVA as the template. According to the Ramachandran plot (see Fig. 2B), there is no residues fell in the disallowed region for the poplar CCoAMOT model, fully proving the rationality of the modeling results.

### Acquisition of ccoaomt** and ccoaomt**_CCoA systems

**Fig. 3:**
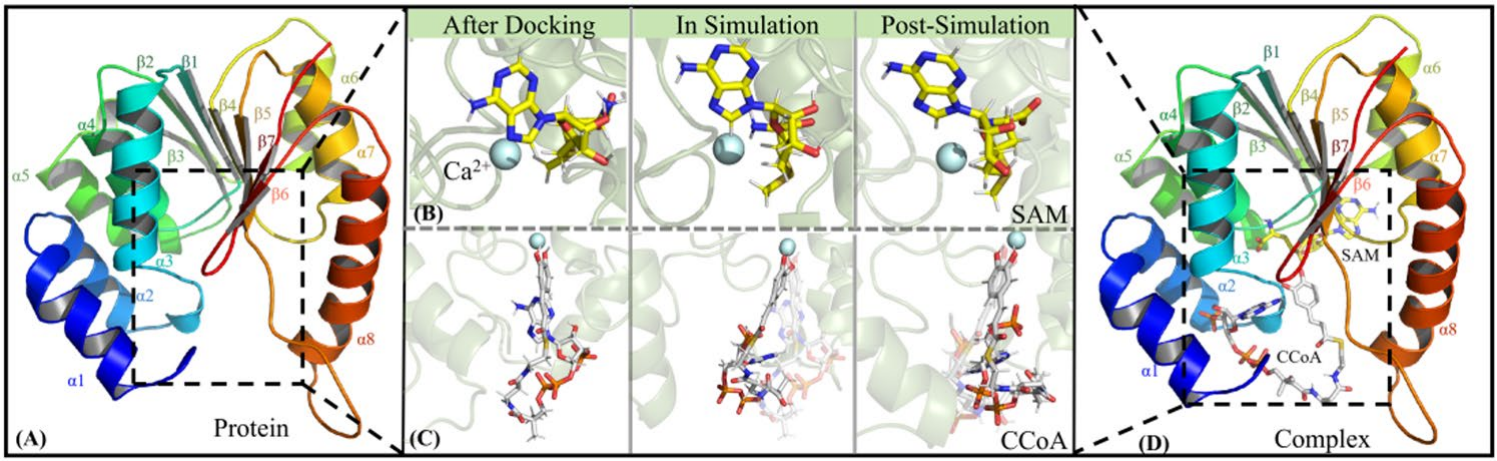
Binding patterns of CCoAOMT sequentially with SAM and CCoA. (**A**) The 3D structure of CCoAOMT; Comparison of bind patterns of SAM (**B**) and CCoA (**C**) with CCoAOMT at different MD stages; (**D**) The refined complex model of CCoAOMT binding with double substrates after MD simulations.

On the basis of the poplar CCoAMOT model above, two complex systems of CCoAOMT binding to SAM (i.e., CCoAOMT*) and further binding to CCoA (i.e., CCoAOMT*_CCoA) were obtained through molecular docking experiments. Both CCoA and SAM are bonded to the Ca^2+^ center of the receptor (see Figs. 3B and C), where the caffeoyl group of CCoA can be coordinated with Ca^2+^ ion via its hydroxyl group. According to Alexander’s site-directed mutagenesis experiments, the Ca^2+^ ion promotes, deprotonation of CCoA by coordinating its active hydroxyl group^26^. In addition, SAM is located in the cavity enclosed by the β1, β2, α4, and α6 regions, and the activation of CCoAOMT confers structural specificity for subsequent CCoA binding to some extent (see Fig. 3D) ^26^. At the secondary structural level, CCoAOMT consists of 8 α-helices, 7 β-sheets and some loops. Among them, the α1, α2, α3 and α8 helices are located inside the active pocket surrounded by a series of dense loops, which effectively prevents the leakage of CCoA. With the sequential binding to SAM and CCoA, the helicity of α1, α2 and α6 in CCoAOMT still maintains stable enough being conducive to structural rigidity of the active pocket, which ensures high efficiency of catalytic reaction (see Fig. 3A).

### Charge and hydrophobicity matching is the prerequisite of substrate binding

**Fig. 4:**
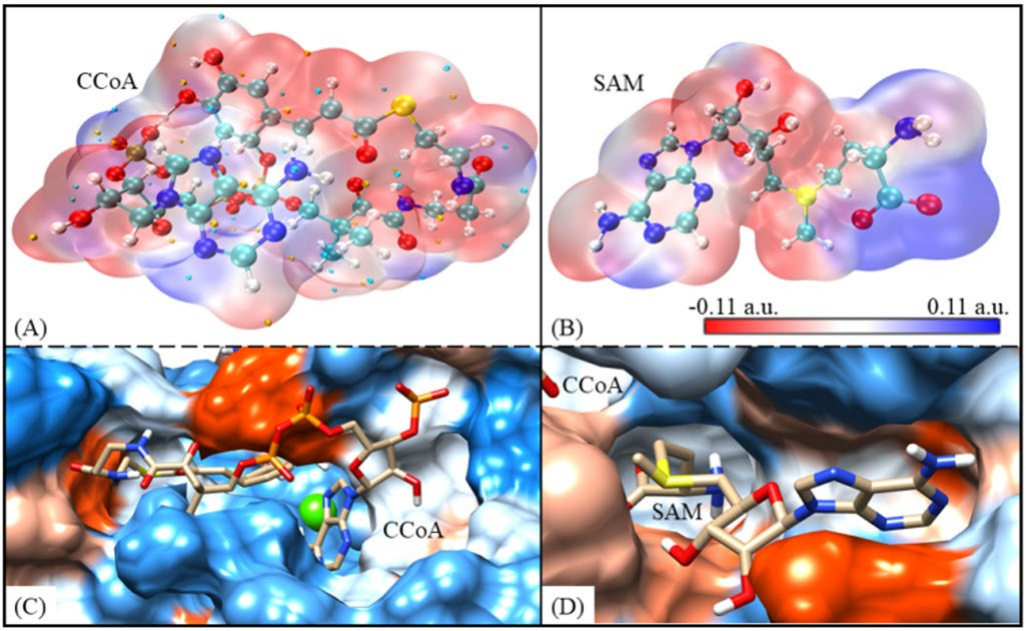
The mean electrostatic potential surface of CCoA (**A**) and SAM (**B**), as well as the interface hydrophobic characteristics of CCoAOMT recognized by CCoA (**C**) and SAM (**D**) in the CCoAOMT*_CCoA system, respectively.

As a double-substrate methyltransferase, CCoAOMT is bound to both CCoA and SAM relying on good charge and hydrophobicity matching. Figure 4 shows the mean electrostatic potential surface of CCoA and SAM, as well as the interface hydrophobic characteristics of CCoAOMT recognized by the two substrates in the CCoAOMT*_CCoA system, respectively. The electrostatic potential surface of CCoA is negatively charged colored in red as a whole, and only a small fraction shows the tendency to take on a positive charge (see Fig. 4A). For SAM, the whole molecule is electrically neutral, except for the middle part being slightly negatively charged (see Fig. 4B). The pKa value at the CCoA binding site was predicted to be 38.5, showing a strong alkaline environment and effectively matching the acidic CCoA. Similarly, the SAM binding cavity of CCoAOMT has a total pKa of 4.7, which tends to bind uncharged molecules.

In addition, the receptor CCoAOMT also has high hydrophobic compatibility with substrates CCoA and SAM, respectively. CCoA is a hydrophilic molecule with polyhydroxyl and polyamino groups, which coincides with the fact that the corresponding pocket exhibits a good hydrophilic environment colored in blue (see Fig. 4C). The SAM binding site of CCoAOMT has no obvious tendency to be hydrophilic, which perfectly matches the ligands with both hydrophobic aromatic rings and hydrophilic hydroxyl/amido groups (see Fig. 4D).

In sum, both CCoA and SAM are well matched to the receptor CCoAOMT in terms of charge and hydrophobic properties. It not only proves the rationality of double substrate recognition sites, but also lays a good structural foundation for subsequent molecular recognition and reactions dominated by CCoAOMT cavities.

### The convergence of trajectories ensures the reliability of molecular recognition and dynamics analyses

The potential energy of the CCoAOMT* and CCoAOMT*_CCoA systems both reach equilibrium after 5 ns, converging to −1.18 × 10^5^ kcal/mol (see Figure S1A). The CCoAOMT*_CCoA trajectory reaches equilibrium earlier than that of CCoAOMT*, and the RMSD value remains at 0.16 ± 0.03 nm, being smaller than the latter’s 0.20 ± 0.05 nm (see Figure S1B). It indicates that the binding of CCoA partially reduces the flexibility of the receptor, resulting in more restrained global motion. The potential energy and RMSD value with small fluctuation both show the reliability of MD trajectories for the CCoAOMT* and CCoAOMT*_CCoA systems.

As shown from Figure S1C, the β4 (D138-V142) and β5 (G163-N170) regions located in the Ca^2+^ center both possess low flexibility, which is conducive to supporting the overall structure and stabilizing the catalytic pocket. The α6 (A120-H134), α8 (R186-P207) regions around catalytic pocket exhibit coordinated high flexibility, while the outmost α1 (D7-V18), α2 (E24-M41) ones show expected high value. The high correlation (*R* = 0.98) of calculated RMSF values between the CCoAOMT* and CCoAOMT*_CCoA systems re-proves the rationality of MD trajectories, which ensures the reliability of the following molecular recognition and dynamics analyses.

### The association with CCoA results in the closure of substrate entry channel

**Fig. 5:**
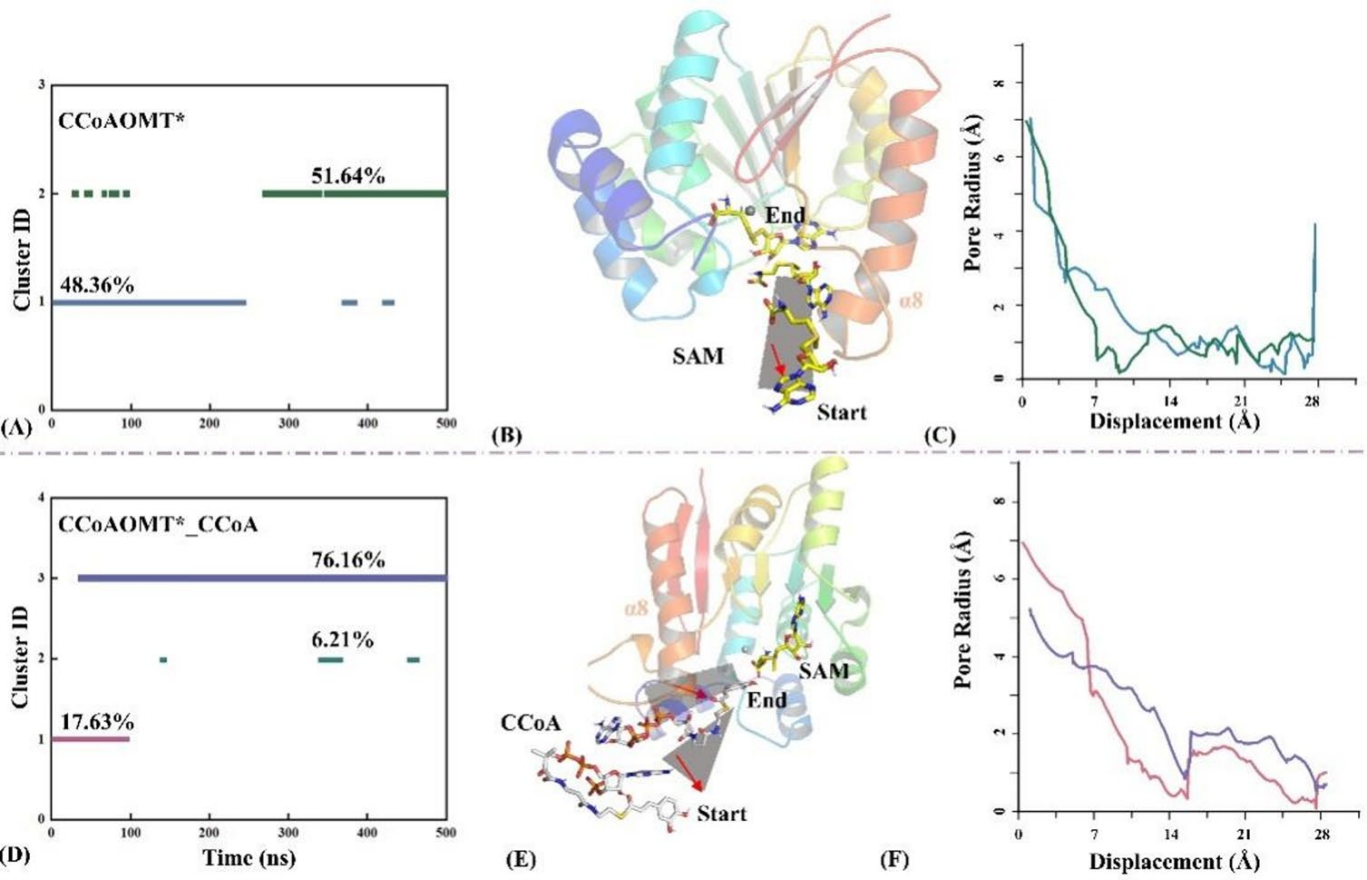
Conformation cluster analyses (**A, D**), representative structural superposition (**B, E**), and radius distribution of substrate entry channels along pore axis (**C, F**) for the CCoAOMT* (**A, B, C**) and CCoAOMT*_CCoA (**D, E, F**) systems.

Conformation cluster analyses were performed for the CCoAOMT* and CCoAOMT*_CCoA systems, with the RMSD threshold value set at 1.7 Å. As shown from Figs. 5A and D, the CCoAOMT* and CCoAOMT*_CCoA systems contain 2 and 3 representative conformation clusters, respectively. In the CCoAOMT* system, the α8 domain undergoes the most frequent conformational changes for two representative snapshots. With the elapse of simulation time, its conformation is gradually stabilized at the open state, being conducive to the subsequent entry and binding of CCoA. Although the CCoAOMT*_CCoA system has three relatively independent representative conformation cluster, the most stable one accounts for overwhelming around 80% snapshots.

Figures 5B and E show the representative structural superposition obtained from conformation clustering for the two systems. Compare with the CCoAOMT* system, the largest conformational changes in CCoAOMT*_CCoA are still distributed in the α8 region. In addition, the substrate transport channel of the latter system changes from the open to closed state, with its structure gradually becoming compact. Figures 5C and F show radius distribution of substrate transport channel along the pore axis for the two systems. The CCoAOMT* system possesses almost constant channel radius of 7 Å, while that in CCoAOMT*_CCoA gradually decreases to 5 Å favoring stable association with CCoA and also preventing its leakage.

### The entry of SAM and CCoA are respectively regulated by W38 and A34

The above conformation clustering analysis showed that the structure of CCoAOMT changed from relatively loose to gradually compact with the binding of CCoA, accompanied by the closure of substrate entry channel simultaneously. Nevertheless, the dynamics characteristics and key regulatory residues of CCoAOMT both remain unclear during the recognition process by two substrates SAM and CCoA. Here, relying on ASMD simulation, the effect of substrate binding on the conformation of CCoAOMT entry channels was quantitatively analyzed.

**Fig. 6:**
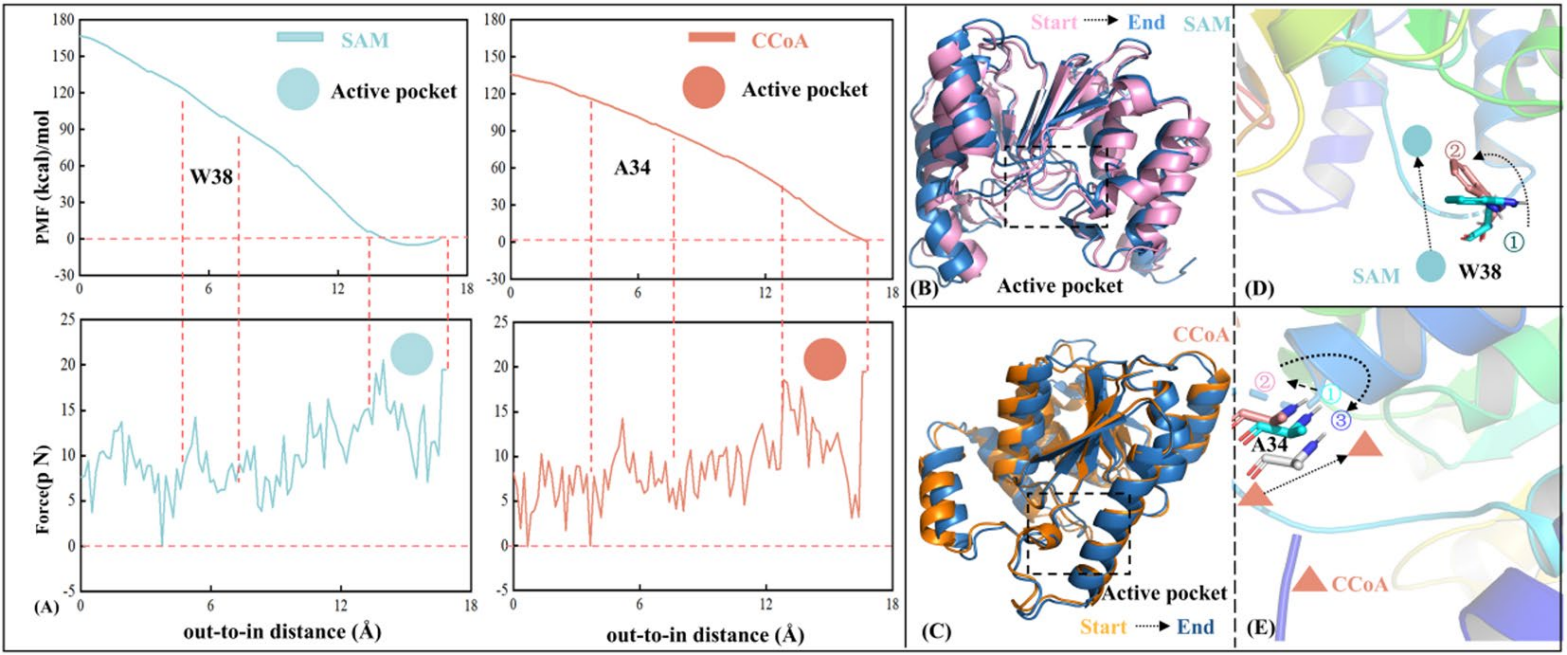
Effect of association with SAM and CCoA on the conformation of substrate entry channel. (**A**) Variations of PMF and traction force with the translocation process for the CCoAOMT* and CCoAOMT*_CCoA systems; Structural superposition before and after binding of SAM (**B**) and CCoA (**C**) by the receptor sequentially; The entry of SAM (**D**) and CCoA (**E**) are respectively regulated by W38 and A34.

Figure 6 shows the variation of mean force potential (PMF) and traction force on two substrates for the CCoAOMT* and CCoAOMT*_CCoA systems, as well as the hindering effect of regulatory residues during the sequential entry process of SAM and CCoA. As shown from Fig. 6A, the entry of SAM from membrane outside to the active pocket requires a smaller PMF, which changes when encountering W38. The indole ring of W38 obstructs the entry pathway for SAM (see Fig. 6D), when the channel radius reaches a minimum accompanied by the maximum traction force. When SAM is fully embedded in the active pocket and bound stably to the receptor, the corresponding PMF is negative, which indicates that SAM has the ability to move freely.

Similarly, CCoA is about to enter the active pocket with a regulatory residue of A34, experiencing maximum collision and large side-chain swing. According to structural superposition, the CCoA entry channel is opened after the association of CCoAOMT with SAM (see Fig. 6B). Next, CCoA can smoothly enter the active pocket as a result. And then, the entry channel becomes closer after substrate binding to prevent the leakage of CCoA. Simultaneously, the receptor underwent a conformational transition from a compact state to a loose intermediate, followed by re-compaction (Fig. 6C). This observation is consistent with the conformation clustering analysis, demonstrating that the CCoAOMT* system transitions from a relatively loose to a compact structure upon substrate (CCoA) binding.

Figure 6D shows the detailed conformational change of W38 during SAM recognition. When SAM approaches the entry channel (i.e., state ① in Fig. 6D), the indole ring of W38 is perpendicular to the channel inside, which is not conducive to its following binding. As SAM continues to move inward (i.e., state ② in Fig. 6D), the indole ring rotates and gradually becomes parallel to the channel, which ensures the entry channel being open. Figure 6E shows the conformation state of the regulatory residue A34 in the recognition and entering process of CCoA, showing significant translational motion (for more detailed information, please refer to Figure S2 A). Specifically, when CCoA approaches the entry channel (i.e., state ① in Fig. 6E), A34 begins to move outwards. With the continuous movement of CCoA, the displacement of A34 reaches the maximum value (i.e., state ② in Fig. 6E), accompanied by a maximization of the radius of entry channel. Finally, CCoA is stably chelated with the active pocket (i.e., state ③ in Fig. 6E), and the entry channel becomes closed.

Furthermore, during the recognition and entering process of CCoA, Y188 located on α8 undergoes a series of coordinated conformational changes to facilitate stable substrate binding. Specifically, when malonyl-CoA begins to approach the active pocket (i.e., state ① in Figure S2 B), its aromatic ring is close to the center of the binding channel, which is unfavorable for the subsequent binding of CCoA. As CCoA gradually enters the pocket (i.e., state ② in Figure S2 B), the aromatic ring of Y188 begins to rotate slowly, adjusting its spatial position to ensure that CCoA can enter the binding channel and prepare for the formation of interactions. When CCoA reaches the catalytic center (i.e., state ③ in Figure S2 B), the aromatic ring of Y188 continues to move to the left, opening the binding channel, ensuring that CCoA is positioned at the correct location, thereby ensuring the accuracy and efficiency of the subsequent catalytic reaction.

On the whole, the conformation of CCoAOMT appears to change from tight to relaxed, and then to tight. In detail, the regulatory residues W38 and A34 may dominate the open-closed state of two entry channels, intelligently ensuring the effective binding and release of two substrates (i.e., SAM and CCoA).

### Closure of entry channel favoring association with substrates and also preventing the leakage of CCoA

**Fig. 7:**
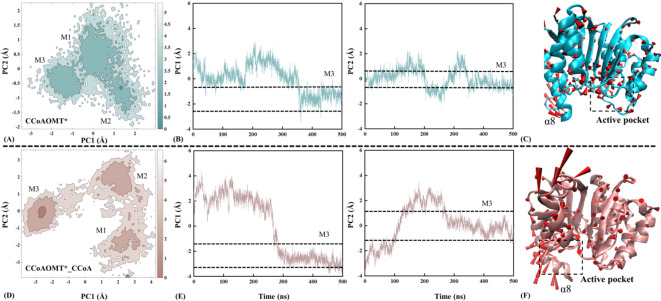
Free energy landscapes (**A, D**), principal component conformational fluctuations (**B, E**) and functional motion modes (**C, F**) for the CCoAOMT* (**A-C**) and CCoAOMT*_CCoA (**D-F**) systems. In panels A and D, the darker the color, the lower the folding free energy. In panels C and F, the arrows and lengths of red cones indicate motion direction and magnitude of the receptor, respectively.

The RMSD analysis above (see Figure S1B) roughly indicates that the binding of CCoA reduces global flexibility of CCoAOMT*_CCoA compared to CCoAOMT*. In the subsequent free energy landscape (FEL) analysis, functional motion modes and conformational changes of the two systems both will be visually revealed, by observing transition among the most stable states. Figure 7 shows FEL, principal component conformational fluctuations and functional motion modes at 300 K for the CCoAOMT* and CCoAOMT*_CCoA systems.

The CCoAOMT* system contains three low free energy regions (i.e., M1, M2 and M3), corresponding to the trajectories in time periods of 50–150, 200–280 and 365–430 ns (see Fig. 7A). In the CCoAOMT*_CCoA system, there are also three independent low free energy regions (i.e., M1, M2, M3), mainly distributed in the time intervals of 30–110, 150–230 and 290–400 ns (see Fig. 7D). The conformational fluctuation of PC2 is closer to Gaussian distribution than that of PC1, proving reliability of PCA results (see Figs. 7B and E). Compared with CCoAOMT*, CCoAOMT*_CCoA possesses similar motion modes except for the α8 domain having the opposite motion direction with larger amplitude (see Figs. 7C and F). In the CCoAOMT* system, the substrate channel for CCoA maintain a stable open conformation. While in the CCoAOMT*_CCoA system, motion modes of the α8 domain lead to narrow channels, favoring the association with substrates and effectively preventing CCoA leakage. It was also described in the previous plant growth regulation work by Liu et al.^58^.

### Enthalpy-driven spontaneous association of CCoAOMT and CCoA

Binding free energy is a widely-used essential index to evaluate the activity of drug molecules, effectively characterizing receptor-ligand affinity and acquiring its recognition driving force. Table 1 lists the contribution of each energy term to the binding free energy between CCoA and CCoAOMT*. When CCoAOMT* combines with CCoA to form a complex, molecular internal energy (i.e., *ELE*_IN_ + *VDW*_IN_) decreases, which is related to the acceptor-ligand stable chelation. It is found that van der Waals energy (i.e., *VDW*_IN_+*VDW*_PB_) serves as the main driving factor of complex formation, releasing 61.41 kcal·mol^−1^. Compared with CCoAOMT*, the electrostatic energy (i.e., *ELE*_IN_+*ELE*_PB_) of the CCoAOMT*_CCoA system is increased by 24.23 kcal·mol^−1^. The total enthalpy change/entropy change/binding free energy are respectively − 37.18/−27.01/−8.87 kcal·mol^−1^, indicating that the molecular recognition of CCoAOMT* by CCoA is enthalpy-driven spontaneous association. The calculated binding free energy (Δ*G*_cal_, −8.87 kcal·mol^−1^) is very close to the experimental data (Δ*G*_exp_, −10.17 kcal·mol^−1^). It is speculated that the strong affinity may be due to larger molecular weight of CCoA and possible interactions with more key residues.

**Table 1:** Contribution of each energy term to binding free energy between ccoaomt** and CCoA (kcal·mol^−1^).

Systems	ELE_IN_	VDW_IN_	ELE_PB_	VDW_SA_	H	TΔS	ΔG_cal_	ΔG_exp_
CCoAOMT*	−7325.69 ± 10.72	−964.62 ± 13.24	−3044.21 ± 78.80	68.97 ± 0.83	−7422.89 ± 84.19	2627.37 ± 10.99	−8.87	−10.17
**CCoA**	−512.76 ± 13.01	−4.52 ± 5.01	−107.70 ± 12.09	6.44 ± 0.47	−454.19 ± 9.45	104.88 ± 0.56		
CCoAOMT*_CCoA	−7917.25 ± 37.44	−1023.19 ± 16.20	−3048.89 ± 97.55	68.05 ± 0.74	−7914.27 ± 82.30	2705.24 ± 5.83		
**Delta**	−78.8 ± 4.59	−54.05 ± 2.06	103.03 ± 30.84	−7.36 ± 0.39	−37.18 ± 11.34	−27.01 ± 4.59		

**Fig. 8:**
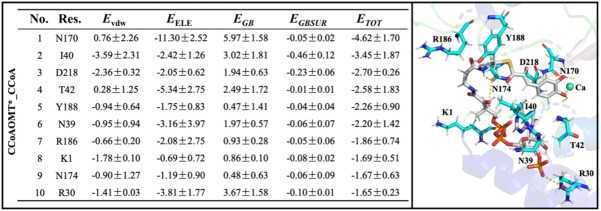
Energy decomposition on 10 key residues (**A**) and receptor-ligand binding pattern (**B**) for the CCoAOMT*_CCoA system. In Panel A, *E*_VDW_ and *E*_ELE_ both are van der Waals energy and electrostatic energy under vacuum, while *E*_GB_ and *E*_GBSUR_ are polar and non-polar parts of solvation effect.

In order to further explore the key residues in recognition process of CCoAOMT* by CCoA, energy decomposition was performed for their binding free energy. Figure 8 shows energy decomposition on 10 key residues of the receptor, along with receptor-ligand binding pattern for the CCoAOMT*_CCoA system. It can be seen that the key residues are all located in the cavity involved in CCoA binding, which is mainly composed of three helixes—α1 (D7-V18), α2 (E24-M41) and α8 (R186-P207). Specifically, CCoA forms H-bonds with the N170/D218/T42/Y188/N39/R186/N174/R30 residues of CCoAOMT*, as well as hydrophobic interactions with K1/I40. It is worth mentioning that Y188/D218 chelates the caffeoyl group of CCoA to the Ca^2+^ active center by forming stable H-bonds, while the ribose part approaches the external solvent region. In sum, the substrate entry channel especially α8 forms H-bonds with CCoA resulting in the overall closure of protein active center, which is conducive to substrate binding and preventing leakage.

### Possible recognition mechanism of CCoAOMT and CCoA

**Fig. 9:**
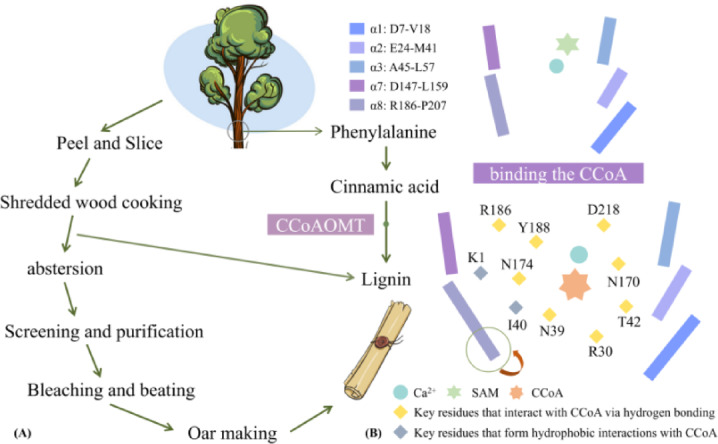
The process of papermaking (**A**), along with possible recognition mechanism (**B**) of CCoAOMT by CCoA.

The papermaking process is composed of peeling and slicing, wood cooking, abstersion, screening and purification, bleaching and beating, pulping in sequence^59^. Chemicals are used in the process of wood cooking and pulping in order to treat unwanted lignin components, thus introducing a large of pollutants. Attention has been paid to the treatment of papermaking pollution, in which reducing lignin content of tree raw material is an important option (see Fig. 9A)^60^. The high enzymatic activity of CCoAOMT is a specific rate-limiting step for the biosynthesis process of lignin in poplar^61^. Therefore, revealing the recognition mechanism of CCoAOMT by CCoA will greatly promote structural design of low-activity CCoAOMT enzymes, being also the core scientific problem of green chemistry in the paper-making industry.

The motion direction and amplitude during the recognition of CCoAOMT by CCoA were given by principal component analysis (PCA). Based on the conformation clustering analysis, the variation of pore radius of substrate entry channel are partially revealed. The calculated results show that the substrate entry entrance of CCoAOMAT is narrowed after binding CCoA, where the functional motion of the α8 helix aids to close the channel preventing substrate leakage. According to the results of energy decomposition, the top 10 key residues for CCoA-CCoAOMT recognition were obtained (see Fig. 9B), all of which ensured structural stability of catalytic center through H-bonds, hydrophobic interactions, as well as coordination with the Ca^2+^ ion.

### Design suggestion for low-activity CCoAOMT

Considering the above molecular recognition, total 200 CCoAOMT mutants were designed by exhaustive mutation on 10 key residues. In our research, the design of 200 CCoAOMT mutants is based on the fundamental principle that each amino acid residue can theoretically be mutated to any of the other 19 natural amino acids (resulting in up to 20 possible variants, including the wild-type in some contexts). For the 10 key residues identified through molecular recognition analysis, we performed exhaustive mutations by substituting each residue with all 19 other natural amino acids, leading to a total of 200 CCoAOMT mutants (including the wild-type for each residue as a reference in our screening process).The selection of low-activity CCoAOMT enzymes followed two core criteria: (1) The basic biological functions should be maintained with its stability difference being not significant (|ΔΔ*G*_folding_| ≤ 2 kcal·mol^−1^), where folding free energies of all CCoAOMT mutants were calculated with FoldX method; (2) The binding free energy of CCoAOMT with CCoA was calculated via SIE method, and the significant increase of corresponding data (ΔΔG_bind_ ≥ 2 kcal·mol^−1^) indicated the decline of specific affinity to CCoA. By finding the snapshot with lowest potential energy through global sampling and fully considering the allosteric effects of proteins, FoldX method is successfully used to calculate the folding free energy with statistical potential function. In fact, FoldX shows a good performance in the discovery of specific skeleton conformation and rotating isomers, agreeing well with experimental data^62^. SIE has been widely used in protein design, by evaluating receptor-ligand affinity to explore signal transduction process^63,64^.

**Fig. 10:**
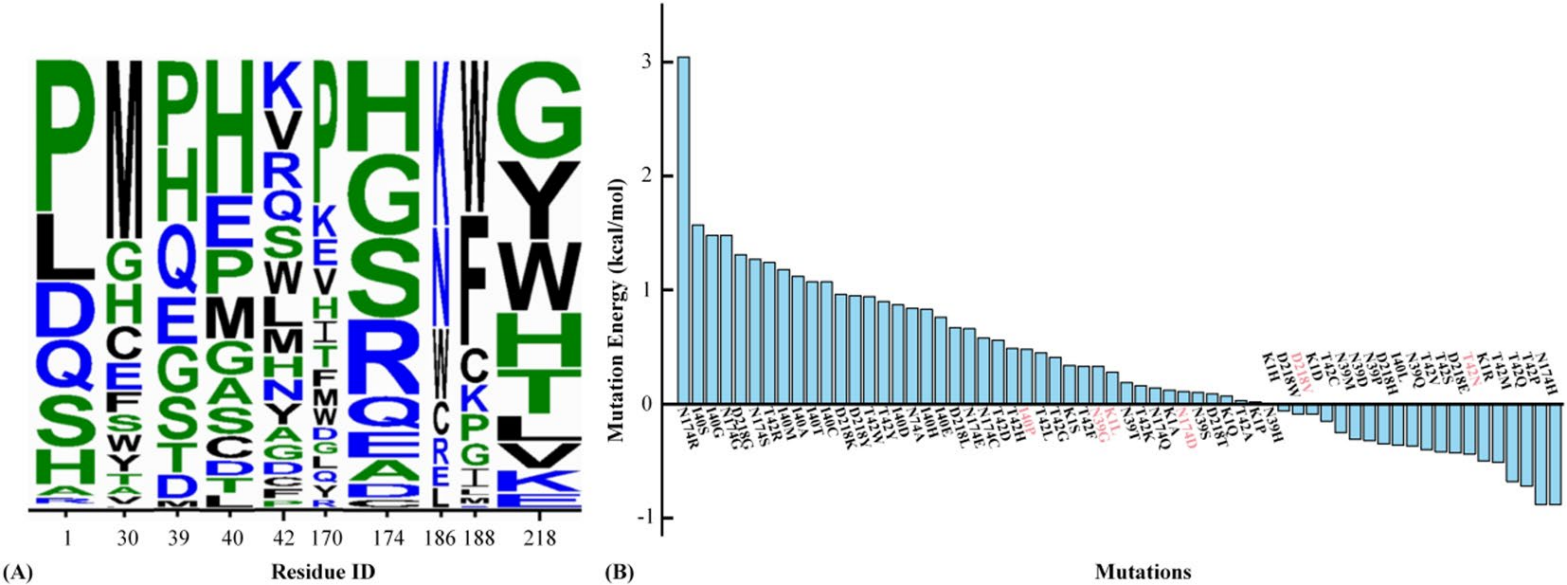
Design suggestion for low-activity CCoAOMT through folding free energy (**A**) and binding energy (**B**) calculation. In panel A, the larger and wider letters correspond to more similar stability to CCoAOMT wild type. In panel B, the pink color is used to mark 6 potential low-activity CCoAOMT mutants.

Structural stability is a prerequisite for enzymes to maintain their biological activity under certain conditions. According to the calculated folding free energy(Δ*G*_folding_), those single mutations at K1/N39/I40/T42/N174/D218 sites were proposed to ensure structural stability of the CCoAOMT, with the alternative number mutations being 8/9/11/17/9/9, respectively (see Fig. 10A). On the premise of ensuring structural stability, 63 mutations on 6 residues were evaluated by the calculation of binding free energy (Δ*G*_bind_). To take into account arbitrariness, six mutants for each residue with higher binding energy were identified, that is, they may correspond to the potentially low-activity CCoAOMT mutants (i.e., K1L/N39G/I40P/T42N/N174D/D218V, see Fig. 10B).

**Fig. 11:**
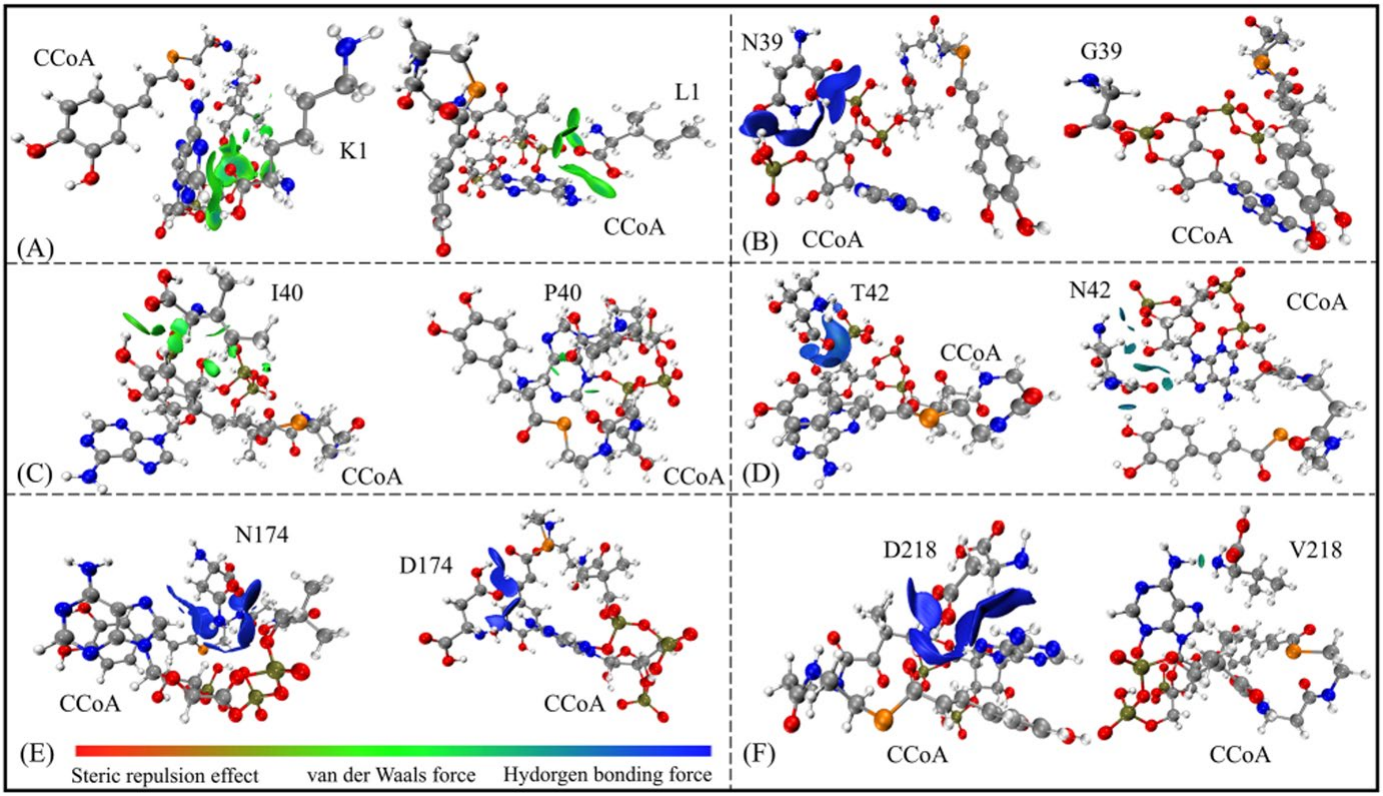
Weak interactions of CCoA in the six CCoAOMT* mutants: K1L(**A**), N39G(B), I40P(**C**), T42N (**D**), N174D(**E**), D218V(**F**). The magnitude of the force values is indicated by different colors and areas on the RDG isosurfaces. Specifically, larger areas indicate stronger forces; blue, green and red colors are used to indicate H-bond forces, van der Waals forces and steric hindrance effect, respectively.

In order to assess the performance of the potentially low-activity CCoAOMT above, SIE method was then used to calculate binding free energies (Δ*G*_bind_) with substrate CCoA (see Table 2). It is found that binding free energies of mutants were higher than that of wild-type (i.e., −10.17 kcal·mol^−1^, see Table 1), which is consistent with the criterion of low activity CCoAOMT. The six mutation sites are located near the CCoA binding pocket, still maintaining a certain level of H-bond and hydrophobic interactions (see Fig. 11).

**Table 2:** Binding free energy of CCoAOMT mutant with substrate CCoA calculated by SIE method (kcal·mol^−1^).

Mutants	ΔGR bind	GR com	GR pro	GR lig	E_vdw_	E_c_	ΔMSA	ΔG_bind_
**K1L**	52.45	−1109.19	−1120.62	−41.01	−54.68	−16.27	−1043.38	−6.24
**N39G**	62.87	−989.36	−1025.40	−26.83	−62.35	−16.10	−966.47	−5.83
**I40P**	50.21	−992.24	−1005.96	−36.48	−53.26	−30.56	−1019.20	−6.43
**T42N**	75.47	−1017.62	−1057.10	−35.99	−69.25	−28.65	−990.20	−6.58
**N174D**	68.49	−1004.28	−1045.56	−27.22	−70.49	−29.81	−903.56	−7.44
**D218V**	52.11	−1104.56	−1117.32	−39.35	−40.36	−20.35	−970.43	−5.10

Specifically, although the T42N and N174D mutants still form H-bonds with the caffeoyl group of CCoAOMT, the variation in side-chain length and H-bond participation atoms partially reduces substrate affinity under the premise of ensuring basic biological functions. In the ribose side K1L/N39G/I40P/D218V mutants, CCoA’s hydrophobic interactions with K1/I40 and H-bonds formation with N39/D218 both were significantly weakened (see Fig. 11). It is worth mentioning that N39 and D218 stabilizes the chelation of CCoA to Ca^2+^ by H-bond, while the corresponding one in the N39G and D218V mutants almost disappear.

## Conclusion

Molecular recognition, conformational change and motion characteristics of CCoAOMT and CCoA were explored via a series of molecular simulation methods, and then potentially low-activity CCoAOMT mutants were designed and determined with free energy-driven strategy. RMSD and FEL analyses showed that the binding of CCoA weakened the flexibility of the CCoAOMT system, and enhanced the structural stability. According to PCA and ASMD analyses, the binding of SAM increases the radius of entry channel facilitating its subsequent recognition by CCoA. In the ternary CCoAOMT complex with SAM and CCoA, the α8 helix is able to reduce the radius of substrate entry channel, which helps to stabilize the CCoA recognition and prevents leakage. In addition, the β4 and β5 regions located in the Ca^2+^ center aid to maintain global structural rigidity of CCoAOMT, while the α1, α2, α6 and α8 regions on the outside of active pocket show high flexibility facilitating the specific recognition of CCoA.

The calculation of binding free energy show that molecular recognition of CCoAOMT by CCoA belongs to the enthalpy-driven spontaneous process, with key driving factor being van der Waals interactions. The key residues closely related to substrate recognition were mined through energy decomposition. Among them, eight residues (i.e., N170, D218, T42, Y188, N39, R186, N174 and R30) formed H-bonds with CCoA, and two (i.e., K1 and I40) formed hydrophobic interactions. Finally, through the prediction of folding free energy, binding free energy and weak interactions, it is proposed that the 6 single mutants (i.e., K1L, N39G, I40P, T42N, N174D and D218V) may be candidates for low-activity CCoAOMT. In addition, our team is further conducting breeding work on mutant poplars, aiming to obtain gene mutation types that can effectively reduce lignin content. This work not only revealed molecular recognition and conformational changes of CCoAOMT and CCoA having certain theoretical significance, but also successfully designed a series of potentially low-activity CCoAOMT mutants showing certain application value for subsequent gene breeding.

## Supplementary Information

Below is the link to the electronic supplementary material.

Supplementary Material 1

## Data Availability

All data generated or analyzed during this study are included in this published article.
