# Supplementary material for: Conformational switch upon substrate binding informs the rational design of CCoAOMT enzymes
Source: Sci Rep. 2025 Oct 21;15:36552. doi: 10.1038/s41598-025-19623-1 (PMC12540674; doi:10.1038/s41598-025-19623-1)
Supplement: Supplementary file 1 — Supplementary Material 1 [file 41598_2025_19623_MOESM1_ESM.docx]

Conformational Switch upon Substrate Binding Informs the Rational Design of CCoAOMT Enzymes

Yujie Cao^1, 2, †^，Xinru Yue^2, †^，Wentong Yu^2^，Pujing Yi^3^, Yuxin Xiao^2^, Liang Ma^2^, Kaixuan Hu^2^, Xin Fu^1^，Jianping Hu^2^，Xiang Nong^1,*^, Wei Liu^1,*^

^1^ School of Life Science, Leshan Normal University, Leshan 614004, China;

^2^ Key Laboratory of Medicinal and Edible Plants Resources Development of Sichuan Education Department, School of Pharmacy, Chengdu University, Chengdu 610106, China

^3^ College of Food Science and Pharmacy, Xinjiang Agricultural University, Urumqi 830052, China

^*^ Correspondence: liuweicdu@sina.com (W.L.); nongx2008@163.com ( X.N.)

^†^ These authors contributed equally to this work.


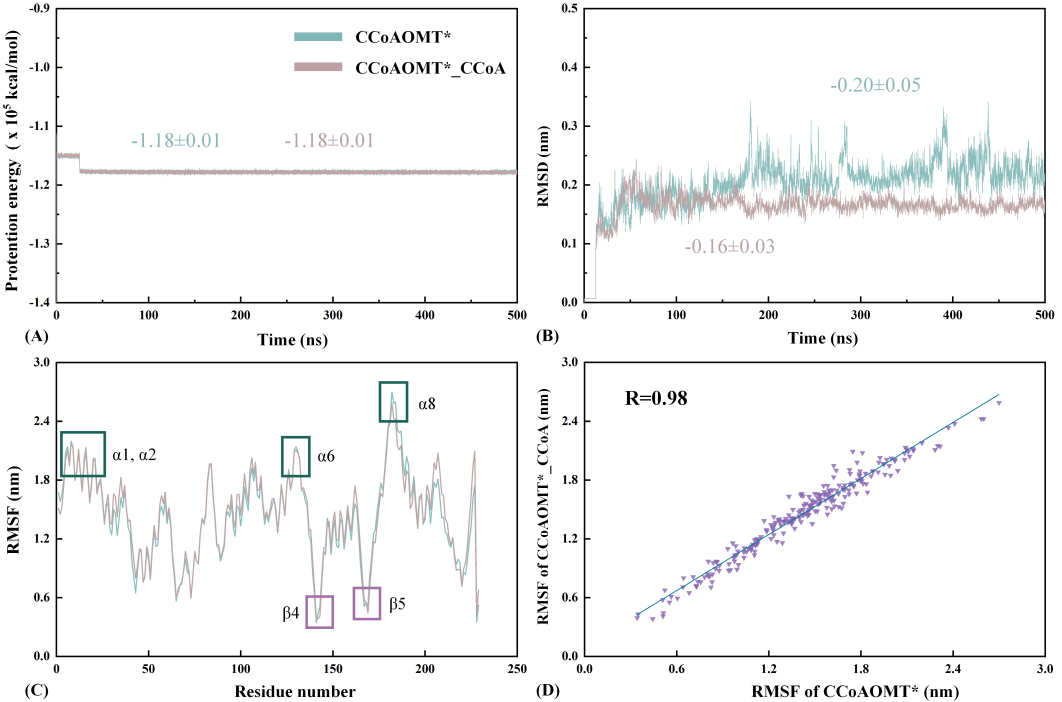


**Figure S1**. Convergence of MD trajectories for the CCoAOMT* and CCoAOMT*_CCoA systems. The variation of potential energy (A) and RMSD (B) values with simulation time, along with RMSF distribution (C) and its correlation (D) for the receptor at residue level.


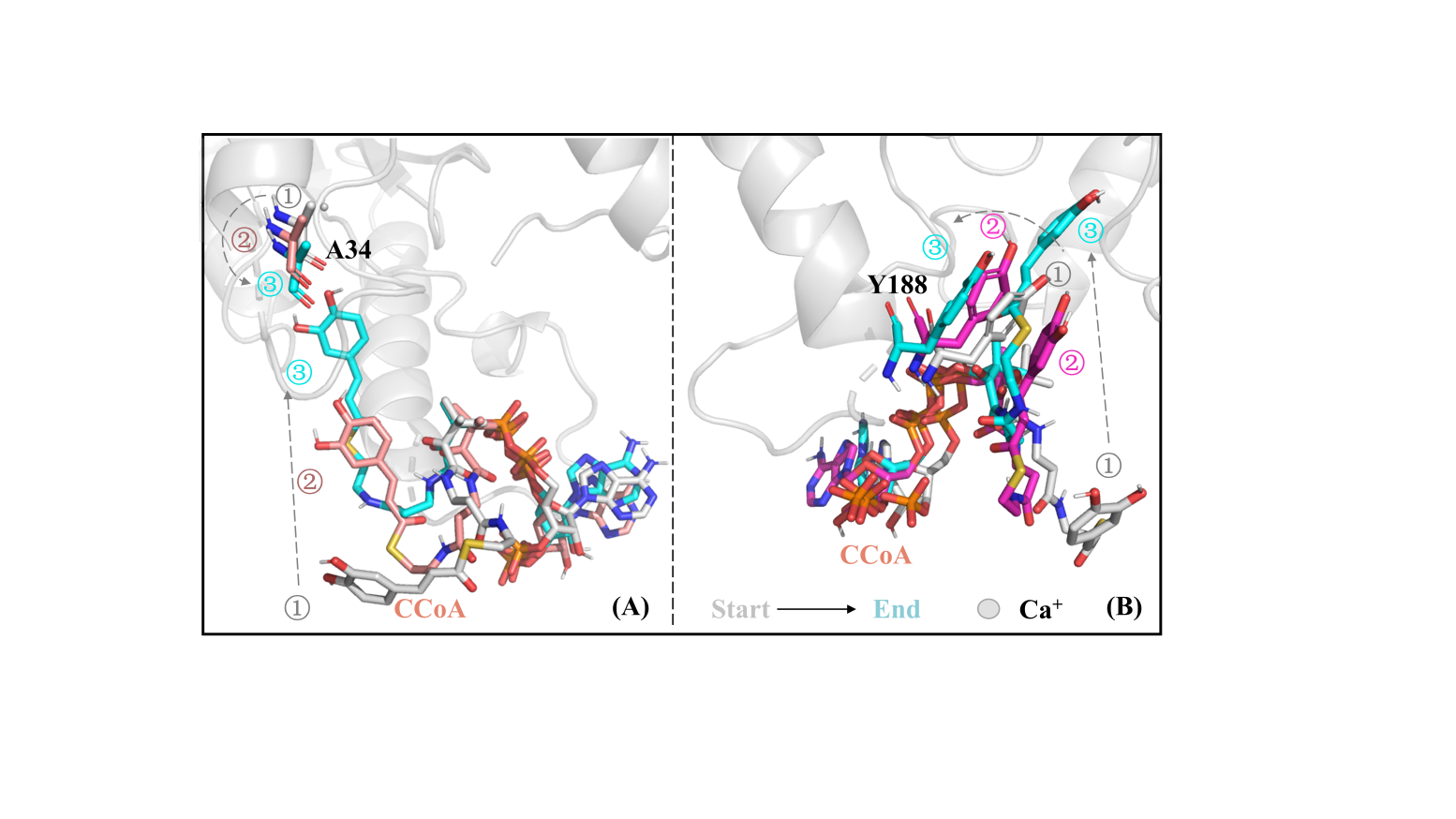


**Figure S2**. During the identification and entering process of CCoA, the coordinated conformational changes of A34 and Y188 occurred.
